# Assessment of Ordinary Kriging and Inverse Distance Weighting Methods for Modeling Chromium and Cadmium Soil Pollution in E-Waste Sites in Douala, Cameroon

**DOI:** 10.5696/2156-9614-10.26.200605

**Published:** 2020-05-04

**Authors:** Romaric Emmanuel Ouabo, Abimbola Y. Sangodoyin, Mary B. Ogundiran

**Affiliations:** 1 Environmental Management, Pan African University Life and Earth Sciences Institute, University of Ibadan, Nigeria.; 2 Department of Agriculture and Environmental Engineering, Faculty of Technology, University of Ibadan, Nigeria.; 3 Department of Chemistry, Faculty of Sciences, University of Ibadan, Nigeria.

**Keywords:** Douala, IDW, interpolation, ordinary kriging, e-waste

## Abstract

**Background.:**

Several studies have demonstrated that chromium (Cr) and cadmium (Cd) have adverse impacts on the environment and human health. These elements are present in electronic waste (e-waste) recycling sites. Several interpolation methods have been used to evaluate geographical impacts on humans and the environment.

**Objectives.:**

The aim of the present paper is to compare the accuracy of inverse distance weighting (IDW) and ordinary kriging (OK) in topsoil analysis of e-waste recycling sites in Douala, Cameroon.

**Methods.:**

Selecting the proper spatial interpolation method is crucial for carrying out surface analysis. Ordinary kriging and IDW are interpolation methods used for spatial analysis and surface mapping. Two sets of samples were used and compared. The performances of interpolation methods were evaluated and compared using cross-validation.

**Results.:**

The results showed that the OK method performed better than IDW prediction for the spatial distribution of Cr, but the two interpolation methods had the same result for Cd (in the first set of samples). Results from Kolmogorov-Smirnov and Shapiro-Wilk tests showed that the data were normally distributed in the study area. The p value (0.302 and 0.773) was greater than 0.05 for Cr and for Cd (0.267 and 0.712). In the second set of samples, the OK method results (for Cd and Cr) were greatly diminished and the concentrations dropped, looking more like an average on the maps. However, the IDW interpolation gave a better representation of the concentration of Cd and Cr on the maps of the study area. For the second set of samples, OK and IDW for Cd and Cr had more similar results, especially in terms of root mean square error (RMSE).

**Conclusions.:**

Many parameters were better identified from the RMSE statistic obtained from cross-validation after exhaustive testing. Inverse distance weighting appeared more adequate in limited urban areas.

**Competing Interests.:**

The authors declare no competing financial interests

## Introduction

Soil is an important part of the urban ecosystem that directly and indirectly affects general quality of life.^1^ The study of spatial distribution and identification of contaminated urban soils and geographical areas with clear identification of source contamination is important for planning and decision making.^2^,^3^ Several studies have identified high variability in soils in urban areas.^4^,^5^ The use of spatial interpolations in geostatistics is increasingly required to understand and solve soil pollution problems in urban areas. Geostatistics describes patterns of spatial data. It provides an estimate and quantitative mapping of the pollution distribution with minimum variance.^6^ A number of factors can affect map quality. These include soil variability, as well as the sampling and interpolation method employed.^7^ Spatial interpolation techniques such as ordinary kriging (OK) and inverse distance weighted (IDW) are widely applied in soil geochemistry for the production of spatial distribution maps of soil parameters.^8^,^3^ Geostatistical methods can provide reliable estimates in locations that have not been sampled.^9^,^10^ Spatial prediction techniques, also known as spatial interpolation techniques, differ from classical modeling approaches as they incorporate information on the geographic position of the sample data points.^11^ The most common interpolation techniques evaluate the estimate for a property at any given location by a weighted average of nearby data.^12^

Questions on the appropriateness of various interpolation methods have evolved, as there is a wide range of these methods. Several comparative studies of relative accuracy have been performed for soil quality parameters. The methods used include geostatistical kriging-based techniques and the IDW method for deterministic interpolation.^13^ Both methods rely on the similarity of nearby sample points to estimate values and create a surface. Deterministic techniques use mathematical functions for interpolation. Geostatistics relies on both statistical and mathematical methods, which can be used to create surfaces and assess the credibility of the predictions. From a theoretical standpoint, OK is the optimal interpolation method.^14^ However, the correct application of OK requires an accurate determination of the spatial structure via semivariogram construction and model-fitting.^12^ Many studies have compared IDW and kriging to evaluate the best prediction method. In some cases, the performance of OK was reported to be better than IDW.^14^,^15^,^16^,^17^,^18^ Research was conducted by Laslett *et al.* to evaluate some interpolation methods for estimation of surface soil pH.^19^ They applied IDW and kriging methods over data obtained from digital elevation models and climate for estimations. They found the kriging method for interpolation to be the most suitable method.^20^ Dayani *et al*. and Hooker and Nathanail used the simple kriging estimator for mapping the pollution of heavy metals in order to estimate the concentration of lead in unsampled areas using the OK error maps to evaluate and control the error of the predicted map at unsampled locations.^21^,^22^

In other studies, IDW generally out-performed OK.^13^ The best results for mapping soil organic matter contents and soil nitrate levels were obtained with the IDW interpolation technique.^23^ The results have often been mixed.^24^,^25^,^26^

Given the variability of results obtained by previous studies, the present study aims to identify the best method for environmental and human health risk evaluation in specific locations in urban areas. This will be done by determining the spatial variability of selected heavy metals in the soil; assessing the accuracy of the IDW and OK interpolation techniques for mapping chromium (Cr) and cadmium (Cd) in the soil of electronic waste (e-waste) recycling sites in Douala, Cameroon; and identifying the spatial prediction method that best illustrates the spatial variability of the environmental and human risk exposure of certain heavy metals. This will enable the identification of the best method for areas where people are at greatest risk with the most urgent need for remediation measures.

**Abbreviations**
e-waste: Electronic waste
IDW: Inverse distance weighting
OK: Ordinary kriging
RMSE: Root mean square error

## Methods

The study area is located in Douala, Cameroon. Douala city is situated at latitude 4°1′north and longitude 9^°^45′ east, on the Wouri estuary, approximately 50 km from the opening of the estuary into the Atlantic Ocean. Douala is divided into 6 districts governed by councils, where a mayor is elected by those councils to preside over the city. The city currently houses about 3.5 million people in a nucleated settlement pattern.^27^ The central area of the city, Akwa, has banks, commercial enterprises and other small-scale businesses. The topography surrounding Douala is characterized by a gentle slope from an altitude of approximately 57 m in the east to approximately 3 m along the Wouri River in the west.^27^ The sloping topography and high run-off rate in the Wouri estuarine system cause groundwater levels in the city to be shallow and above the soil surface in some areas. The city is consequently subjected to frequent severe floods almost year-round. This applies in particular to Mabanda and Bonendale in Bonaberi to the north and the Youpwe area in the south, as illustrated by Guevart *et al*. in a computer-generated view of the flood prone areas.^27^

Ngodi, Makea and New Bell are hotspots with the highest concentration of e-waste recycling activities in Douala. They are located in the town and are surrounded by other economic activities.

### Sampling

A total of 30 soil samples, 10 per area, were collected from Makea, Ngodi and New Bell. Collection took place in the first half of 2017. The samples were collected with the aid of a soil auger at a depth of 5 to 15 cm, representing the topsoil. Each point of sample collection was recorded with a global positioning system with a precision error of ±3 m. This was used to obtain the altitude, longitude, and latitude figures of the locations, which were later imported into a geographic information system (ArcGIS 10.1). The samples were stored in aluminum foil before they were transferred into plastic bags and bottles. As the collection was done randomly, the recycling sites were not uniformly distributed.

The samples were analyzed with an atomic absorption spectrometer. A representative sample was first pulverized using a mortar and pestle. About 1 g of each sample was weighed into the dry digesting tube. A total of 5 ml of concentrated perchloric acid was added in the ratio and stirred. The digesting tube was placed in a water bath set at 100°C and boiled for 2 hours. To avoid caking, the sample was shaken vigorously at 20-minute intervals and the resulting solution, referred to as stock solution, was filtered and made up to 50 ml with distilled water, and stored in polyethylene bottles prior to instrumental analysis. The stock solution was used to directly determine the elements (Cr and Cd) with an atomic absorption spectrometer. The distribution of the points is shown in Figure 2. The slope degree and aspect were measured with the processing of an Advanced Spaceborne Thermal Emission and Reflection Radiometer image.

**Figure 1:**
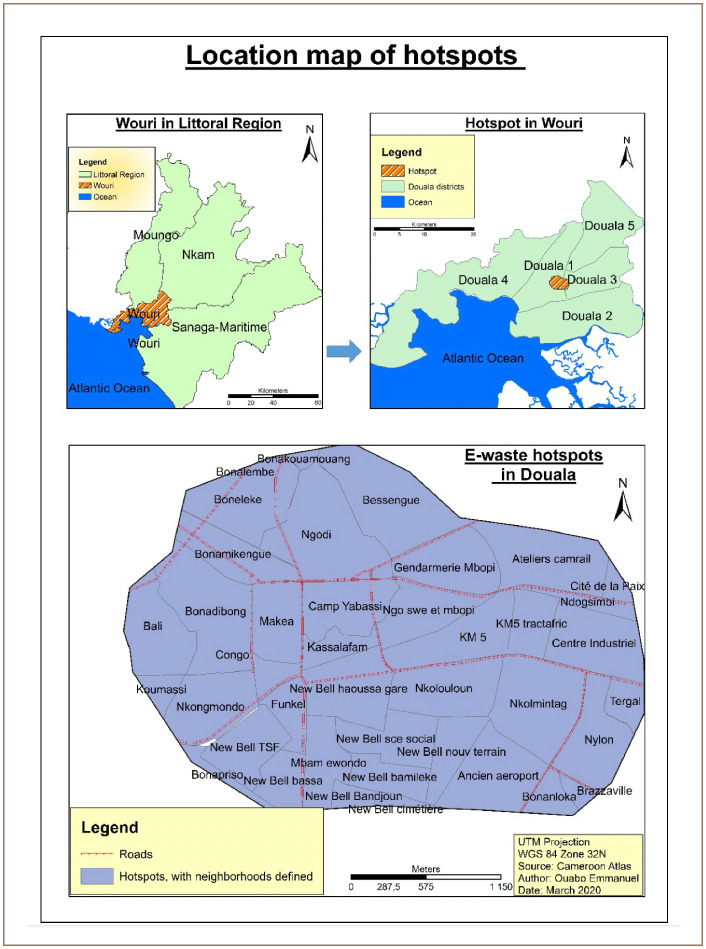
Location map of the hotspot in Douala

**Figure 2:**
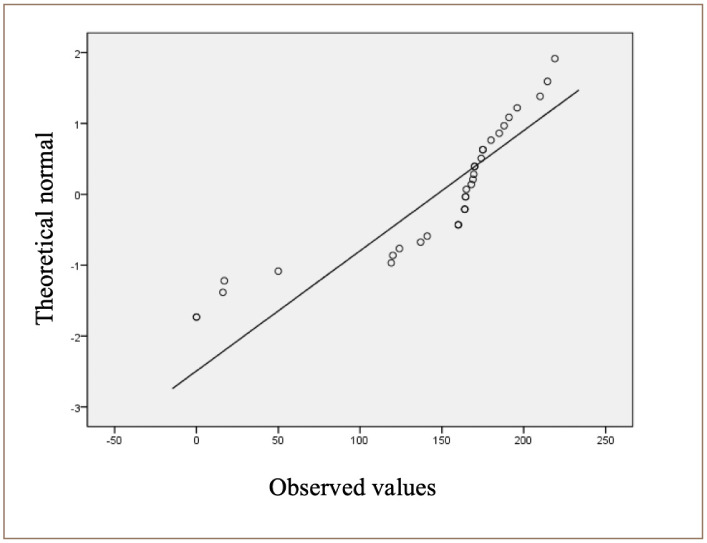
Tests of normality for chromium

### Spatial and statistical methods

The hierarchical Bayesian Poisson mixture model includes a prior distribution for the unknown parameters in the environment as well as the data distribution. The Poisson distribution is a model commonly used to count data. The Poisson distribution assumes that the variance and mean of the data are equal. This is not the case for the data that were used in this study. However, it is a good instrument that allows inference and estimation using software and a mixed model.

### Hotspot analysis

Hotspot analysis is both a statistical and spatial method used in estimating hotspot concentration of particular elements or features. In the field of spatial interpolation, the Hot Spot Analysis tool (from ArcGIS) calculates the Getis-Ord Gi* statistic for each feature in a dataset. The resultant z-scores and p-values identify where features with either high or low values cluster in space. This tool works by looking at each feature within the context of neighboring features. A feature with a high value is interesting, but may not be a statistically significant hotspot. To be a statistically significant hotspot, a feature will have a high value and be surrounded by other features with high values as well. One of its assumptions is that the collected points should be equally distributed, with the same distance between each point collected;^9^ which was not the case in the field.^28^

### Geoadditive models, spatially adaptive models and mixed models

Introduced by Kammann and Wand, geoadditive models analyze the spatial distribution of the variables while accounting for possible non-linear covariate effects.^29^ Moreover, the geoadditive model can be extended to include generalized responses, small area estimation, longitudinal data, missing data, and so on.^30^ It gained popularity in applied research as a flexible and interpretable regression technique because it maintains the assumption of additivity of the covariates effects, allowing the presence of nonlinear relationships with the response variable.^31^

The linear mixed model representation is a useful instrument because it allows estimation using mixed model methodology and software. They represent such effects by merging an additive model that accounts for the non-linear relationship between the variables, and that accounts for the spatial correlation by expressing both as a linear mixed model.^32^

### Geostatistical analysis

There are two main categories of interpolation methods. The deterministic interpolation techniques (IDW, radial basis functions, global polynomial interpolation) that use mathematical functions such as Euclidean distance and the family of geostatistics which relies on both mathematical and statistical models that take positive autocorrelation into account.^33^ The present study compared the OK and IDW methods for Cr and Cd soil interpolation.

### Ordinary kriging and inverse distance weighting methods

Ordinary kriging and IDW are the most popular spatial interpolation methods used in the field of environmental studies. Both methods have been developed based on the assumption that nearby points have more correlations and similarities than distant observations. The IDW interpolation is largely a reflection of Waldo Tobler's first law in geography which states that “everything is related to everything else, but near things are more related than distant things”.^34^ The IDW method assumes that the distance between neighbors is proportional to the similarities and the rate of correlations between them; this is defined as a distance reverse function of every point from nearby points.^35^ The IDW method works best with evenly distributed points in an area. It is possible to control the significance of known points upon the interpolated values, based on their distance from the output point with IDW. The weights for samples in IDW decrease with an increase in distance between the known samples and the estimated points. These weights are controlled by weighting powers, so that greater powers reduce the effect of farther estimated points and smaller powers distribute the weights more uniformly among the neighbors' points. Inverse distance weighting does not make assumptions about spatial relationships except for the basic assumption that nearby points will be more closely related than distant points to the value at the interpolated location.

The main factors affecting the accuracy of IDW interpolation are the “rate” of decreasing weight (defined as the power parameter of distance ‘p'), the size of the neighborhood and the number of neighbors which are relevant for the accuracy of results.

Inverse distance weighting was calculated using the Equation 1.^36^

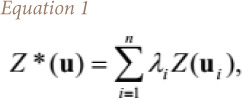
where, u is the estimation location, , , , 1 , u_i_, i = 1,…., n, are the locations of the sample points within the neighborhood, Z*(u) is the inverse distance estimate at the estimation location, n is the number of sample points, λ_i_, _i_ = 1,…, n, are the weights assigned to each sample point, and Z(u_i_)_, i_ = 1,…, n, are the conditioning data at sample points. The weights are determined using Equation 2:

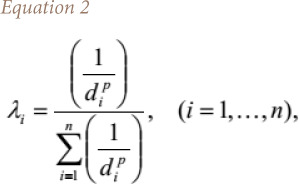
where, d_i_ are the Euclidian distances between estimation location and sample points and exponent p is the power or distance exponent value. Note that the sum of the inverse distance weights λ_i_, _i_ = 1,…, n, is equal to 1, that is *(Equation 3)*:

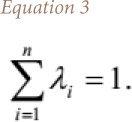



The value applied for the power p varies generally from 1 to 4 and are commonly recommended. One of the main factors affecting the accuracy of the IDW is the value of the power parameter.

Ordinary kriging provides an estimate of an unsampled point based on the weighted average of observed neighboring points within a specific area. At the difference of IDW, the OK method is not deterministic but extends the proximity weighting approach of IDW to incorporate random components where the exact point is unknown. The weights in OK are decided based on the spatial structure parameters of a variogram which measures the relationships between squared differences between paired samples and their distances.^8^ The OK method samples input data and models the relationship between the variance in value and distance between points. The spatial autocorrelation between measured sample points was examined using semivariogram/covariance. Anisotropy semivariogram was used to model the spatial relationship in the dataset and to find the best fit model that passes through the points in the semivariogram.^9^ In order to estimate the spatial and statistical relationship as well as perform the interpolation and calculate the surface, the OK method used the semivariogram.

The OK equation is outlined in Equation 4:

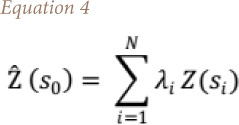
where, Ẑ (s0) is the predicted location, λ_i_ is the unknown weight of the measured value of pairs of points at _i_th location, Z (s_i_) is the measured value of pairs of points at _i_th location, and N is the number of measured values of pairs of points multiplied by the distance h.

The normality of data was tested using the Kolmogorov-Smirnov and Shapiro-Wilk normality test. These tests are used to detect sample of data normality from a population with a specific distribution.

When performing IDW and OK, cross validation is part of the output of the Geostatistical Analyst Toolbar results in ArcGIS 10.1. Cross validation provides summary measures of error and allows comparison between interpolation methods. The parameters used for the comparison are the mean error and root mean square error (RMSE). The mean error is the average difference between observed and predicted values. The RMSE shows how well the model predicts observed values. Low values for both error statistics indicate a better model.^37^

The mean error is used to determine the degree of bias in the estimates and is calculated using Equation 5.

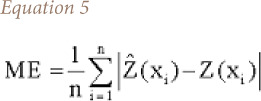
where, ME is the mean error. The RMSE provides a measure of the error size that is it sensitive to outliers. The RMSE value can be calculated with Equation 6.

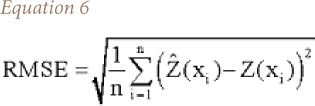
The mean absolute error provides an absolute measure of the size of the error. Mean absolute error is calculated with Equation 7.

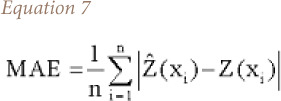
where, MAE is the mean absolute error.

The mean error gives an indication of how well the data value fits into the neighborhood of the surrounding values. It is the result of the difference between the measured value and the estimated cross validation. The closer the average of the cross validation error is to zero, the error decreases; there is no apparent bias. Overestimation and underestimation of the model is indicated by a positive and negative bias, respectively. Thus, the mean standardized error is the mean error divided by the standard deviation where the mean error value depends on the data scale. The value of the mean standardized error should be as close to zero as possible. For an optimum result, the predictions should be as close to the measured values as possible. To assess the accuracy of the method, the RMSE is very useful; it indicates how closely the model predicts the measured values. The estimates are more accurate as long as the RMSE is close to zero. The average standard error is calculated to evaluate the deviation from the observation. If this value is close to zero, then the deviation from the observation is minimal.^38^

The variability in prediction is correctly assessed if the average standard error is close to the root-mean-squared prediction error. If the average standard error is greater than the root-mean-squared prediction error, the variability of the predictions is overestimated and vice versa. Therefore, the result of each prediction error divided by its estimated prediction standard error should be similar. Thus, if the prediction standard error is valid, the root-mean-square standardized error should be close to one. Greater than one root-mean-square standardized error values indicate an underestimation and less than one indicates that the prediction errors are overestimated.^39^,^40^

### Geographic information system mapping procedure

The IDW and OK of the spatial analyst extension in ArcGIS 10.1 was used in the mapping of variables. All the measured points (Cr and Cd) were used in the calculation of each interpolated cell based on the results of the laboratory analysis of each sample. Thus, 15 points were removed randomly from the current data points from their original analysis. Then a test was conducted to see which method better predicts the concentrations in the samples that were left out. A feature dataset (Douala shapefile) was used for the mask. The legend classification was based on the concentration levels of pollutants in the hotspot.

## Results

Table 1 shows the Cd and Cr concentrations that were measured in the laboratory. Each is attached to a particular location with global positioning system points. The values presented in Table 1 were used for the interpolations.

**Table 1:** Cadmium and Chromium Concentration in Selected Dumpsites, Douala, Cameroon

**Sample no**	**Cd value mg/l**	**Cr value mg/l**	**Sample no**	**Cd value mg/l**	**Cr value mg/l**	**Sample no**	**Cd value mg/l**	**Cr value mg/l**
Tests 21*	10	20						
Tests 25*	10	20						
NGO 1	30	90	MAK 1	20	140	NB 1	10	80
NGO 2	20	8	MAK 2	20	130	NB2	20	100
NGO 3	20	40	MAK 3	20	80	NB3	30	40
NGO 4	30	10	MAK 4	20	150	NB4	20	80
NGO 5	30	80	MAK 5	20	130	NB 5	20	40
NGO 6	30	40	MAK 6	20	200	NB6	30	140
NGO 7	30	130	MAK 7	20	190	NB7	20	90
NGO 8	30	70	MAK 8	20	80	NB 8	30	90
NGO 9	25	130	MAK 9	20	90	NB9	30	20
NGO 10	20	30	MAK 10	10	90	NB 10	30	20

*control sites

Abbreviations: S, site: NGO, Ngodi; MAK, Makea; NB, New Bell

Test site 21 and 25 are samples of the topsoil from a natural environment (control). As can be seen, there was a significant difference in concentrations between control samples and e-waste recycling sites samples.

One assumption of the geostatistical analysis is that the data have to be normally distributed. In order to assess the normality of data, the Kolmogorov-Smirnov and Shapiro-Wilk tests were applied. The results *(Tables 2 and 3)* showed that the data distributions were a good fit. The calculated p-values were greater than 0.05 and the data points were distributed around the line.

**Table 2:** Tests of Normality for Chromium

	**Kolmogorov-Smirnov^a^**			**Shapiro-Wilk**		

	**Statistics**	**ddl**	**Signification**	**Statistics**	**ddl**	**Signification**
Cr value mg/l	0.302	35	0.000	0.773	35	0.000

^a^ Significance correction of Lilliefors

Abbreviation: ddl, Degree of freedom

**Table 3:** Tests of Normality for Cadmium

	**Kolmogorov-Smirnov^a^**			**Shapiro-Wilk**		

	**Statistics**	**ddl**	**Signification**	**Statistics**	**ddl**	**Signification**
Cd value mg/l	0.267	35	0.000	0.712	35	0.000

^a^ Significance correction of Lilliefors

Abbreviation: dll, Degree of freedom

Table 2 gives the result of the Kolmogorov-Smirnov and Shapiro-Wilk normality tests for Cr. For the two tests applied, the data are normally distributed in the study area. The p value (0.302 and 0.773) is greater than 0.05.

### Observed values

Figure 2 shows the distribution of data points around the line, indicating that the data points are normally distributed along the line.

Table 3 gives the result of the normality test of Kolmogorov-Smirnov and Shapiro-Wilk for Cd. For the two tests applied, the data are normally distributed in the study area with p value of 0.267 and 0.712, which is greater than 0.05.

### Observed value

**Figure 3:**
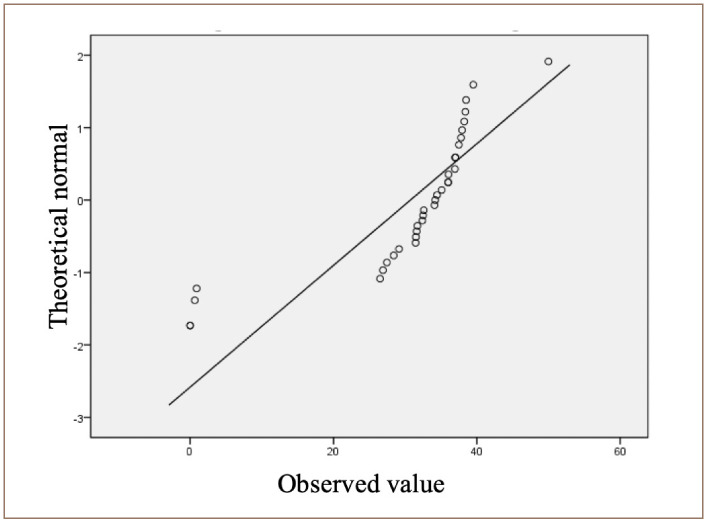
Tests of normality for cadmium

Figure 3 shows the distribution of Cd data points around the line, indicating that the data points are normally distributed along the line and therefore over the study area.

### Results of the interpolated maps comparing the two techniques

**Figure 4:**
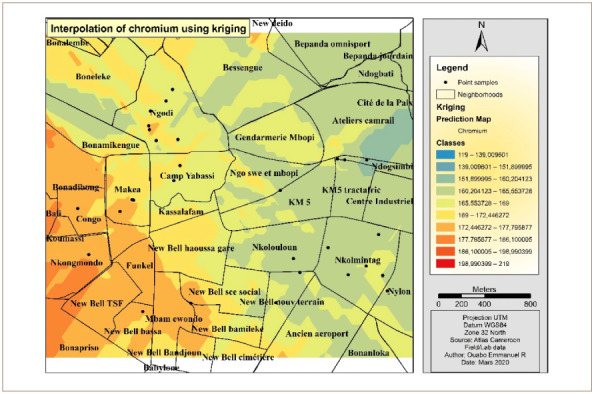
Interpolated map of chromium with the ordinary kriging method

Figure 4 presents the Cr interpolation over the study area. The western and southwestern parts of the hotspot had the highest concentrations of Cr. However, the eastern and the northeastern areas were less polluted by Cr. The OK interpolation method was used to derive this map.

**Figure 5:**
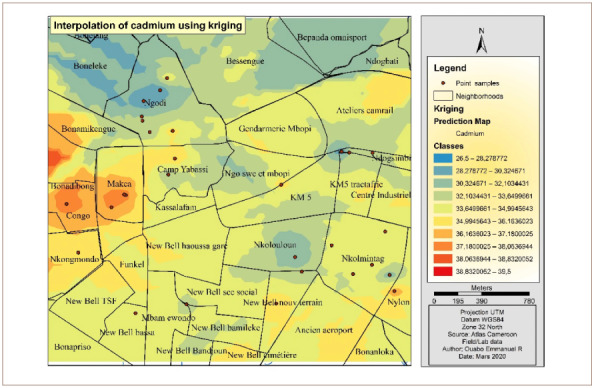
Interpolated map of cadmium with the ordinary kriging method

Figure 5 presents the Cd interpolation over the study area. The western part of the hotspot had the highest concentrations of Cd. The other parts of the study area were less polluted and are represented with the color yellow. Furthermore, the yellow color indicates an average concentration over the main part of the study area, while the northern part had the lowest concentration.

**Figure 6:**
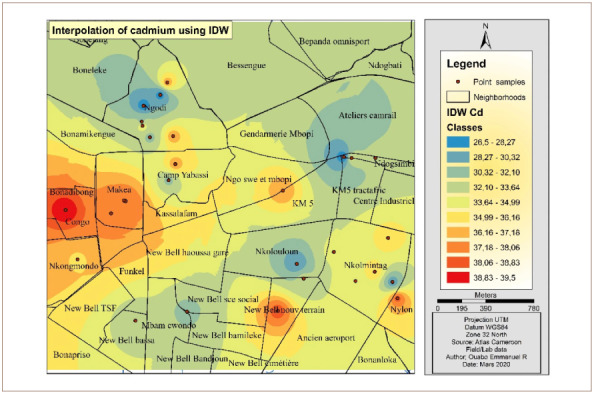
Interpolated map of cadmium with the inverse distance weighting method

Figure 6 presents the IDW Cd interpolation over the study area. The western part of the hotspot had the highest concentrations of Cd. Other parts of the map which were less polluted are represented by the color yellow and are predominantly in the north and northeast.

**Figure 7:**
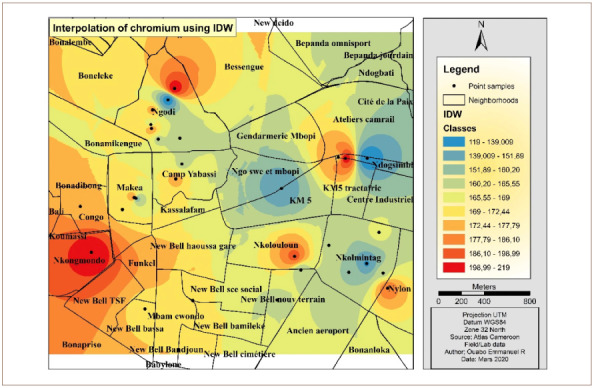
Interpolated map of chromium with the inverse distance weighting method

Figure 7 shows the map of Cr interpolation over the study area. The west, southwest and portions of the north and northwest of the hotspot were the areas with the highest concentration of Cr. The eastern and the northeastern parts were less polluted with Cr.

### Results of the interpolated maps by the two techniques (based on samples left out)

**Figure 8:**
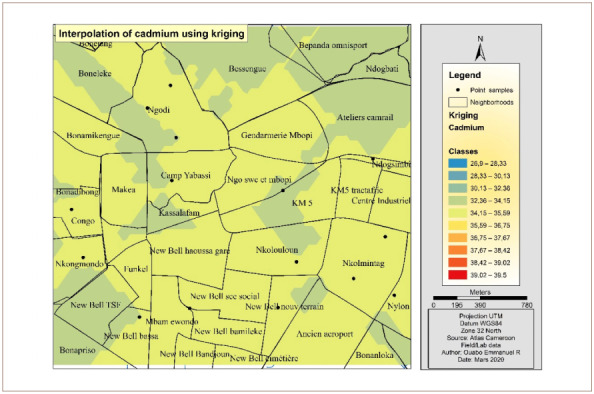
Interpolated map of cadmium with the ordinary kriging method

**Figure 9:**
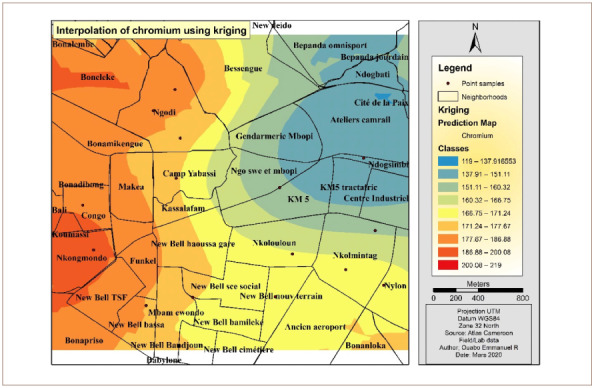
Interpolated map of chromium with the ordinary kriging method

**Figure 10:**
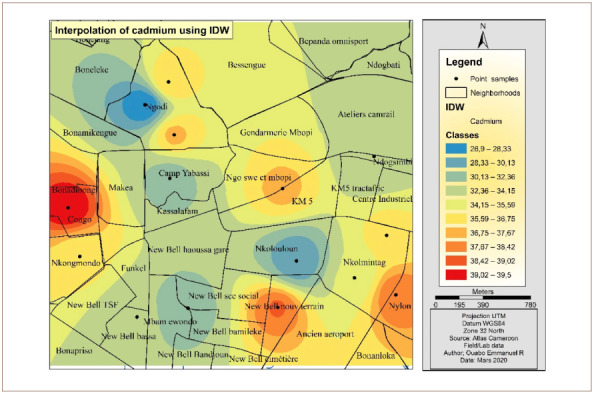
Interpolated map of cadmium with the inverse distance weighting method

**Figure 11:**
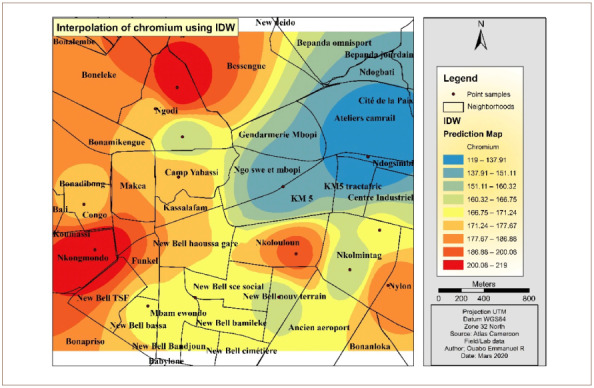
Interpolated map of chromium with the inverse distance weighting method

In order to further assess the performance of the two methods, 15 samples were randomly removed from the initial set of data. Then, the same analyses were performed with samples left out. The results of the interpolated maps are shown in Figures 8–11.

Figure 8 presents cadmium interpolation over the study area. The map has almost a uniform concentration around samples points all over the study area. It appears that the interpolation made a map averaging the concentration of the pollutant.

Figure 9 presents Cr interpolation over the study area. The concentration of Cr noticeably increases from the western to the eastern part of the hotspot.

Figure 10 presents Cd interpolation over the study area. The western and southeastern part of the hotspot had the highest concentration of Cd. However, the northern and the northeastern areas were less polluted by Cd.

Figure 11 shows the map of Cr interpolation over the study area. The west, southwest and portions of the north and northwest had the highest concentrations of Cr. The northeastern parts were less polluted by Cr.

## Discussion

The OK map results provided a comprehensive distribution of pollutants in the study area, with the potential concentration values. The OK results (Cd and Cr) did not fully take into consideration the reality of the urban area in the hotspot. From 1–100 m distance, concentrations varied greatly. However, the IDW maps gave a more reliable representation of the situation. The e-waste recycling sites were relatively small, and therefore the concentrations were not spread over a wide spectrum in the study area. The IDW interpolation gave a better representation of the concentration of Cd and Cr in the maps of the study area.

### Comparison of interpolation performance

For Cr, the comparison of cross validation results with OK and IDW showed varying errors from one point to the other. For the OK method *(Table 4)*, the error ranged from ±1 to ±53, showing that between locations, the error was minimized or maximized, depending on the measured values. In the case of IDW, the error ranged from ±1 to ±73 with a mean error of 2.40, compared to −0.75 obtained in OK.

**Table 4:** Cross Validation Summary of Chromium with Ordinary Kriging

	**Measured**	**Predicted**	**Error**
Maximum	219.00	175.5972	55.46796
Minimum	119.00	157.4815	−53.4602
Mean	168.70	167.9408	−0.75918

The mean of the predicted values was 167.94 for the OK method and 171 for the IDW method *(Table 5)*. Of the two methods, the result from OK was closer to the actual mean of the measured values (168.7).

**Table 5:** Cross Validation Summary of Chromium with Inverse Distance Weighting

	**Measured**	**Predicted**	**Error**
Maximum	219.00	202.7136	73.78169
Minimum	119.00	140.5065	−73.9935
Mean	168.70	171.0687	2.368655

In the case of Cd, the comparison of cross validation results between OK and IDW shows that the error varies from one point to the other. For the OK method *(Table 6)*, the error range of ±1 to ±8.3 shows that the error is minimized or maximized, depending on the measured values. However, the error range was not wide. In the case of IDW, the error ranged from ±1 to ±9.0 with a mean error of −0.40, compared to −0.26 for OK.

**Table 6:** Cross Validation Summary of Cadmium with Ordinary Kriging

	**Measured**	**Predicted**	**Error**
Maximum	39.5	38.0457	8.369907
Minimum	26.5	29.31114	−7.68887
Mean	34.13	33.86917	−0.26083

For Cd interpolation with OK and IDW, the accuracy in the results were similar, with IDW having a slight advantage. In this case, the results were very close to each other, with IDW showing slightly better results compared to OK. The mean of predicted values was 33.86 for the OK method and 33.72 for the IDW method *(Table 7)*. Of the two methods, the result obtained from the OK method was closer to the mean of the measured values, but the IDW method reported a better difference.

**Table 7:** Cross Validation Summary of Cadmium with Inverse Distance Weighting

	**Measured**	**Predicted**	**Error**
Maximum	39.5	38.17815	−9.05455
Minimum	26.5	26.73521	8.054287
Mean	34.13	33.72236	−0.40764

### Validation results: comparison of interpolation performance

**Figure 12:**
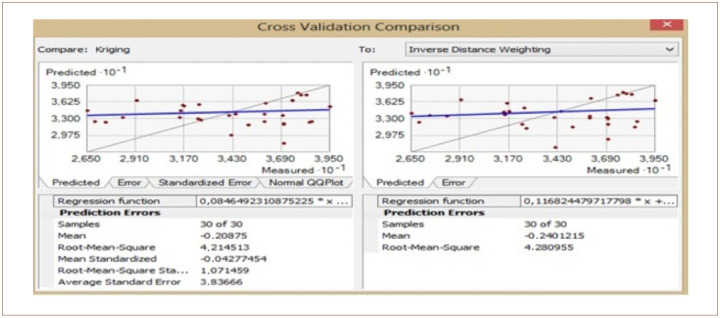
Cross validation comparison of cadmium

The cross-validation comparison of the OK and IDW methods for Cd is shown in Figure 12. The mean value was −0.2 for OK and −0.2 for IDW. The root mean square was 4.21 for OK and 4.28 for IDW. From these two values, it can be concluded that OK and IDW methods can be used interchangeably as both are good for Cd modelling.

For the OK, the standardized mean error recorded was −0.04. This value was very close to zero, showing that the model was good Cd interpolation. The RMSE was 1.07 while the average standard error was 3.83. The OK method produced a good interpolation model for Cd prediction in the study area. It is important to note that the IDW method does not have this information listed in order to be compared with the OK technique. However, the root mean squares are close, showing that the two models are a good fit for Cd.

**Figure 13:**
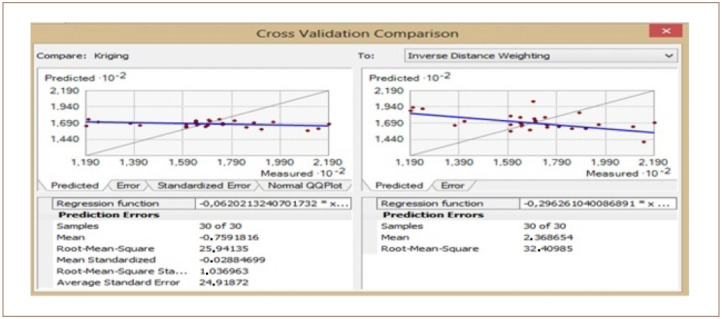
Cross validation comparison of chromium

The cross-validation comparison of the OK and IDW methods for Cr is shown in Figure 13. The mean values were −0.75 and 2.36 for the OK and IDW methods, respectively. The root mean square was 25.94 for OK and 32.40 for IDW. Using these values, the OK method produced a better result compared to IDW for Cr.

For the OK method, the standardized mean error was −0.02, showing that the model was good for Cr interpolation. The root-mean-square error was 1.03 and the average standard error was 24.91. Thus, the OK result produced a better interpolation model for Cr prediction in the study area compared to IDW.

### Comparison of maps results after leaving out samples

After leaving out 15 samples points in order to run the analysis again, The OK and IDW map results provided a comprehensive distribution of pollutants in the study area, with potential concentration values. The OK results (Cd and Cr) were greatly diminished and the concentrations dropped, looking more like an average. This is due to the fact that the included number of samples was smaller compared to the initial set of samples. The IDW interpolation gave a better representation of the concentration of Cd and Cr on the maps of the study area. In the case of Cr, the levels of concentration are more visible with a wider spatial occupation compared to the map with initial samples.

### Comparison of interpolation performance after leaving out samples

For Cr, the comparison of cross validation results with OK and IDW showed varying errors from one point to the other.

For the OK method *(Table 8)*, the error ranged from ±9 to ±13, showing that between locations, the error was minimized or maximized, depending on the measured values.

**Table 8:** Cross Validation Summary of Chromium with Ordinary Kriging

	**Measured**	**Predicted**	**Error**
Maximum	219	188.42	13.03643
Minimum	119	139.21	9.494681
Mean	169	163.81	11.26555

In the case of IDW, the error was 1 with a mean error of 1, compared to 11.26 obtained in OK *(Table 9)*. The error was brought to 1 for IDW, a reduction compared to the initial sample points interpolated.

**Table 9:** Cross Validation Summary of Chromium with Inverse Distance Weighting

	**Measured**	**Predicted**	**Error**
Maximum	219	219	1
Minimum	119	119	1
Mean	169	169	1

For Cd interpolation with OK and IDW, the accuracy was similar to the initial set of samples *(Tables 10 and 11)*. The results were very close to each other.

**Table 10:** Cross Validation Summary of Cadmium with Ordinary Kriging

	**Measured**	**Predicted**	**Error**
Maximum	39.5	34.7083	1.33377
Minimum	26.9	33.9884	1.00824
Mean	33.20	34.215333	1.32286

**Table 11:** Cross Validation Summary of Cadmium with Inverse Distance Weighting

	**Measured**	**Predicted**	**Error**
Maximum	39.5	39.5	1
Minimum	26.9	26.9	1
Mean	33.20	33.20	1

The mean of the predicted values was 34.21 for the OK method and 33.20 for the IDW method *(Tables 10 and 11)*. From the two methods, the results obtained from both were closer to the mean of the measured values, but the IDW reported a smaller difference in terms of error.

**Figure 14:**
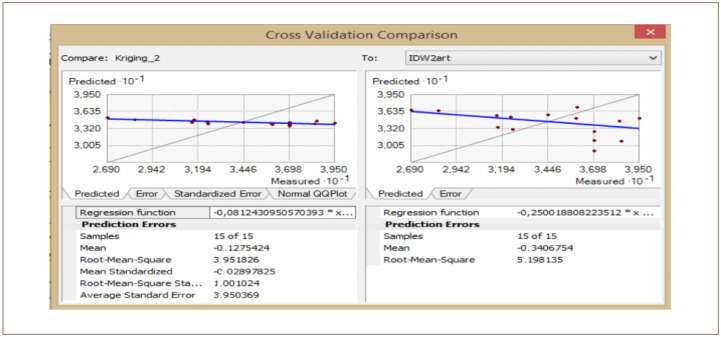
Cross validation comparison of cadmium

The cross-validation comparison of the OK and IDW methods after dropping some of the samples for Cd is shown in Figure 14. The mean value was −0.12 for OK and −0.34 for IDW. The root mean square was 3.95 for OK and 5.19 for IDW. From these two values, it can be concluded that the OK and IDW methods can be used interchangeably as both are good for Cd modelling.

For the OK method, the standardized mean error was −0.02. This value was very close to zero, showing that the model was a good fit for Cd interpolation. The RMSE was 1.001, while the average standard error was 3.95. The OK and IDW methods produced good interpolation models with almost identical results for Cd prediction in the study area.

**Figure 15:**
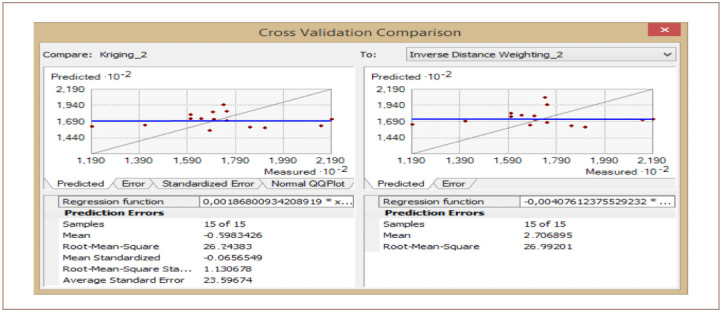
Cross validation comparison of chromium

The cross-validation comparison of the OK and IDW methods for the samples of Cr left out is shown in Figure 15. The mean value was −0.59 for OK and 2.70 for IDW. The root mean square was 26.24 for OK and 26.99 for IDW. These results indicate that the OK and IDW methods can be used interchangeably, as both are good fits for Cr modelling.

For OK, the standardized mean error recorded was -0.06. This value was very close to zero, showing that the model was good for Cr interpolation. The RMSE was 1.130 and the average standard error was 23.59. Based on the mean, the OK method produced a good interpolation model for Cr prediction in the study area with samples left out. However, the root-mean-squared provided very similar results for OK and IDW.

## Conclusions

The present study applied techniques based on fundamental theorems of surfaces to interpolate the spatial patterns of Cd and Cr in soils from e-waste recycling sites. It provided quantitative information on the best interpolation method for heavy metals in e-waste sites located in urban areas. Many studies have looked at the interpolation methods of heavy metals in soils,^45^ but few have conducted a comparison of interpolation methods for heavy metals coming from e-waste recycling sites in urban areas of developing countries. The findings demonstrated that OK had more accurate results compared to IDW for Cr. The OK interpolation method was more accurate than IDW and this aligns with conclusions of several other studies.^41^,^22^,^42^,^43^ Therefore, OK is more suitable for Cr, however not for Cd in urban areas, which was a slight advantage in favor of IDW. Ordinary kriging was considered to have generally superior performance compared to IDW as the prediction error was lower for the OK method. Ordinary kriging was more accurate for Cr, but it was on the same level as IDW for Cd. In order to validate this conclusion, 15 samples were left out and the same analyses were carried out to evaluate the best interpolation method. The OK method showed better results using this technique. However, in urban areas where people are concentrated in a limited space, the results showed IDW to be more accurate in evaluating exposure risk in specific locations. These spatial interpolation methods had various decision parameters. However, by adjusting the power parameter of IDW, a better and smoother result can be obtained. This partly explains its popularity as an interpolation method, especially in evaluating water pollution.^44^ The mean error and RMSE showed that OK was more suitable for Cr, but not for Cd compared to IDW results. Therefore, for interpolation of heavy metals in urban areas, OK is not considered to be the best method for interpolation. It is, however, recommended that the accuracy of the interpolation models be checked by evaluating the error of each model and using the one with the lowest error. These results can assist geographic information system specialists in selecting the best method for assessing pollution levels of heavy metals in e-waste sites.
